# Role of Superoxide Anions in the Redox Changes Affecting the Physiologically Occurring Cu(I)-Glutathione Complex

**DOI:** 10.1155/2011/674149

**Published:** 2011-09-20

**Authors:** Hernán Speisky, Camilo López-Alarcón, Claudio Olea-Azar, Cristian Sandoval-Acuña, Margarita E. Aliaga

**Affiliations:** ^1^Nutrition and Food Technology Institute, University of Chile, Santiago 7830489, Chile; ^2^Faculty of Chemical and Pharmaceutical Sciences, University of Chile, Santiago 8380492, Chile; ^3^Facultad de Química, Pontificia Universidad Católica de Chile, Santiago 6094411, Chile

## Abstract

The physiologically occurring copper-glutathione complex, [Cu(I)-[GSH]_2_], has the ability to react continually with oxygen, generating superoxide anions (O_2_^∙−^). We addressed here the effects that superoxide removal has on the redox state of Cu(I) and GSH present in such complex and assessed the formation of Cu(II)-GSSG as a final oxidation product. In addition, we investigated the potential of a source of O_2_^∙−^ external to the Cu(I)-[GSH]_2_ complex to prevent its oxidation. Removal of O_2_^∙−^ from a Cu(I)-[GSH]_2_-containing solution, whether spontaneous or Tempol-induced, led to time-dependent losses in GSH that were greater than those affecting the metal. The losses in GSH were not accompanied by increments in GSSG but were largely accounted for by the cumulative formation of Cu(II)-GSSG molecules. Notably, the redox changes in Cu(I) and GSH were totally prevented when Cu(I)-[GSH]_2_ was coincubated with hypoxanthine/xanthine oxidase. Data suggest that the generation of O_2_^∙−^ by Cu(I)-[GSH]_2_ implies the obliged formation of an intermediate whose subsequent oxidation into Cu(II)-GSSG or back reduction into Cu(I)-[GSH]_2_ is favoured by either the removal or the addition of O_2_^∙−^, respectively.

## 1. Introduction

The Cu(I)-glutathione complex is believed to be formed within cells during the interaction between Cu^2+^ ions and reduced glutathione (GSH). In fact, following the exposure of either human hepatoma cells (HACs) [1, 2] or intestinal epithelial cells (Caco-2) [3] to copper, most of the metal is recovered bound to GSH molecules, forming, most likely, a Cu(I)-[GSH]_2_ complex. The exact biological role of the latter complex has not been fully established. Some studies suggest, however, that it may play a role as carrier of Cu(I) to copper-dependent enzymes, such as SOD [4], and to copper-storing and copper-transporting proteins, such as metallothionein [5, 6] and ceruloplasmin [7], respectively. 

Studies conducted in noncellular systems indicate that mixing Cu^2+^ ions and GSH in a molar ratio equal to or greater than 1 : 3 leads to the swift formation of Cu(I)-[GSH]_2_ [8–10], as in (Rx. 1)

(Rx. 1) $$2{\text{Cu}}^{2+}+6\text{GSH}\to 2\text{Cu}\left(\text{I}\right)\text{-}{\left[\text{GSH}\right]}_{2}+\text{GSSG}+2{\text{H}}^{+}$$

The formation of the Cu(I)-glutathione complex has been supported by ^1^H-NMR and EPR studies [4, 11] and would involve an initial stoichiometric reduction of Cu^2+^ by GSH and a subsequent chelation of the cuprous ion by two additional GSH molecules [10]. Binding of GSH has been suggested to stabilize Cu(I) in a form that, for long time, was presumed to be redox-inactive due to the relative stability that the complex exhibits in oxygen-containing solutions [4, 12–14]. Contending such concept, we recently provided evidence to support the contention that rather than redox-inactive, the Cu(I)-[GSH]_2_ complex is able to react with molecular oxygen in a reaction which reversibly leads to the formation of superoxide radicals and an “intermediate oxidized complex” (IOC) [15, 16] (Rx. 2)

(Rx. 2) $$\text{Cu}\left(\text{I}\right)\text{-}{\left[\text{GSH}\right]}_{2}+{\text{O}}_{2}⇄\text{IOC}+{{\text{O}}_{2}}^{∙-}$$

An important “exception” to the reversibility of (Rx. 2) would be given by the presence of a superoxide-interceptor in the media. In fact, we recently described [16] that the addition of SOD to a solution containing Cu(I)-[GSH]_2_ leads to the formation of a stable product whose NMR spectrum is identical to that of a preformed Cu(II)-GSSG complex. Thus, removal of superoxide from (Rx. 2) appears to drive the oxidative conversion of IOC into Cu(II)-GSSG in a presumably irreversible manner. In the present study, we have addressed the redox changes that, under conditions leading to superoxide removal, affect the metal and the thiol present in the Cu(I)-[GSH]_2_ complex during its oxidative conversion into Cu(II)-GSSG. In addition, we investigated the potential of a source of superoxide anions, external to (Rx. 2), to prevent the oxidation of Cu(I)-[GSH]_2_ into Cu(II)-GSSG.

## 2. Experimental

### 2.1. Chemicals and Reagents

Cupric chloride (CuCI_2_ · 2H_2_O), reduced glutathione (GSH), oxidized glutathione (GSSG), glutathione reductase (GR; E.C. 1.6.4.2. from baker's yeast), *β*-Nicotinamide adenine dinucleotide 2′-phosphate reduced tetrasodium salt hydrate (NADPH), hypoxanthine (HX), xanthine oxidase (XO; E.C. 1.17.3.2. from bovine milk), bathocuproine disulfonic acid (BCS), and 5-(3-carboxy-4-nitrophenyl)disulfanyl-2-nitrobenzoic acid (DTNB) were all purchased from Sigma-Aldrich. 4-hydroxy-2,2,6,6,-tetramethylpiperidine-1-oxyl (Tempol) was purchased from Calbiochem. EDTA was from Bio-Rad Laboratories. All aqueous solutions were prepared in Chelex-100-treated Tris-buffer (20 mM; pH 7.4).

### 2.2. Preparation of Copper-Glutathione Complexes

The Cu(I)-[GSH]_2_ complex was prepared as previously reported [15], mixing CuCI_2_ and GSH in a 1 : 3 molar ratio. Whenever referring to a given concentration of such complex, it should be understood that it reflects the concentration of copper used in its preparation. The Cu(II)-GSSG complex (there on preformed complex) was prepared by direct mixing of CuCI_2_ and GSSG in a 1 : 1 molar ratio as previously described [17]. Unless indicated otherwise, solutions containing the Cu(I)-[GSH]_2_ and Cu(II)-GSSG complexes were always prepared immediately (90 seconds) before using.

### 2.3. Determination of Thiol-Titratable Groups

Thiol-titratable groups (TTG) were quantified employing DTNB as thiol reacting agent [18, 19]. The increase in OD_412 nm_ associated with the formation of 5′-thio-2-nitrobenzoic acid (TNB) was monitored at 30°C in a 96-well plate using a Multi-Mode Microplate Reader (Synergy HT), as done previously [20]. The assay was initiated by the addition of samples of the Cu(I)-[GSH]_2_ complex (Tempol-treated and control) to a solution containing a mixture of DTNB (24 *μ*M) plus EDTA (2 mM). Results were estimated using a molar absorption coefficient of 14.15 mM^−1^ cm^−1^ [21], and expressed as GSH equivalents. Control experiments were carried out using GSH instead of the complex.

### 2.4. Determination of the Cu(II)-GSSG Complex

The concentration of Cu(II)-GSSG in solutions containing a preformed complex was assessed as described by Postal et al. [17], measuring the optical density at 625 nm in a Unicam He*λ*ios *α* spectrophotometer. Likewise, the formation of such complex within a solution containing a mixture of the Cu(I)-[GSH]_2_ complex plus Tempol (incubated during 0–90 min) was also assessed at 625 nm. Neither Cu^2+^ nor GSH, Cu(I)-[GSH]_2_, or Tempol exhibited significant absorbance at such wavelength. Results were estimated using a molar absorption coefficient of 60 M^−1^ cm^−1^ [17] and expressed as millimolar concentration of Cu(II)-GSSG.

### 2.5. Determination of EDTA-Releasable Oxidized Glutathione

Cu(II)-GSSG was assessed employing the NADPH/glutathione reductase assay, as described by Tietze [22] but in the presence of added EDTA (2 mM). The decay in OD_340 nm_ associated with the formation of NADP^+^ was monitored at 30°C. The assay was initiated after the addition of NADPH, glutathione reductase and EDTA to a cuvette containing either a freshly prepared Cu(I)-[GSH]_2_ complex, a Cu(I)-[GSH]_2_ complex preincubated with Tempol during 90 min, or a preformed Cu(II)-GSSG complex. Results were estimated using a molar absorption coefficient of 6.22 mM^−1^ cm^−1^ for NADPH [23] and expressed as micromolar concentration. As such, they represent the sum of free GSSG plus the GSSG released by the addition of EDTA to a Cu(II)-GSSG complex present in the assay media.

### 2.6. Determination of Cuprous Ions

Cu(I) was assessed as previously described [24], using bathocuproine as Cu(I) chelating agent. The increase in OD_480 nm_ associated the Cu(I)-[BCS]_2_ complex formation which was monitored at 30°C. The assay was initiated by the addition of BCS (500 *μ*M) to samples containing a Cu(I)-[GSH]_2_ complex that had been preincubated (during 0–60 min) in the presence or in the absence of Tempol. Results were estimated using a molar absorption coefficient of 12.25 mM^−1^ cm^−1^ [24], and expressed as micromolar concentrations of Cu(I)-bathocuproine.

### 2.7. Determination of Thiol-Titratable Groups and Cu(I) in a Mixture of Cu(I)-[GSH]_2_ plus Hypoxanthine/Xanthine Oxidase

A Cu(I)-[GSH]_2_ complex was incubated with a mixture of hypoxanthine (HX; 0.2 mM) plus xanthine oxidase (XO; 2 U/mL) during 1–60 min (30°C). Since XO was found to significantly contribute with its own thiols to the TTG assay, its removal was necessary. Thus, mixtures containing the enzyme were centrifuged (4000 ×g, 4°C for 40 min) through Ultrafree-CL tubes (Centrifugal Filter Devices; NMWL 100000, Millipore). Ultrafiltrates thus obtained were subjected to the above-referred TTG, EDTA-releasable GSSG and Cu(I) assays.

### 2.8. Data Expression and Analysis

**Figure 1:**
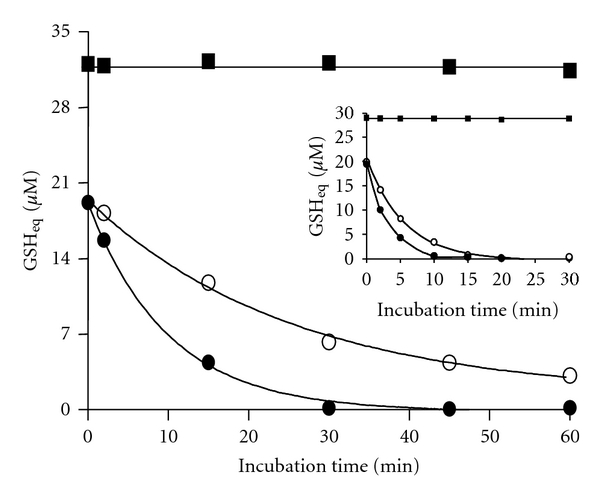
Effect of Tempol on thiol-titratable groups of the Cu(I)-[GSH]_2_ complex. Tempol was added to a solution containing the Cu(I)-[GSH]_2_ complex and the mixture incubated at 30°C for 0–60 min. Samples from such incubation were added DTNB (24 *μ*M) and the increase in OD_412 nm_ registered 5 min later. Results are expressed as GSH equivalents (*μ*M). The symbols represent: (■) GSH (30 *μ*M); (○) Cu(I)-[GSH]_2_ (10 *μ*M); (●) Cu(I)-[GSH]_2_ (10 *μ*M) plus Tempol (8 *μ*M). Insert to the figure shows results obtained from carrying out the same experiment but at 37°C, during 0–30 min.

**Figure 2:**
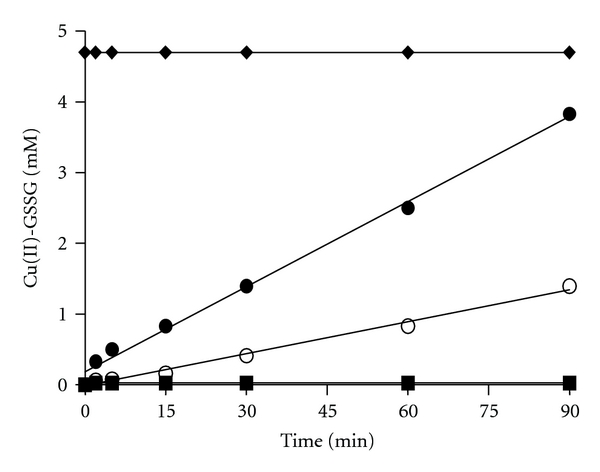
Time-dependent formation of Cu(II)-GSSG after the addition of Tempol to a Cu(I)-[GSH]_2_ complex. Tempol was added to a solution containing the Cu(I)-[GSH]_2_ complex and the mixture incubated at 37°C for 0–90 min. Samples were taken during the incubation and the increase in OD_625 nm_ registered. Results are expressed as mM Cu(II)-GSSG concentration. The symbols represent: (♦) Cu(II)-GSSG (5 mM); (■) GSH (15 mM); (○) Cu(I)-[GSH]_2_ (5 mM); (●) Cu(I)-[GSH]_2_ (5 mM) plus Tempol (4 mM).

**Figure 3:**
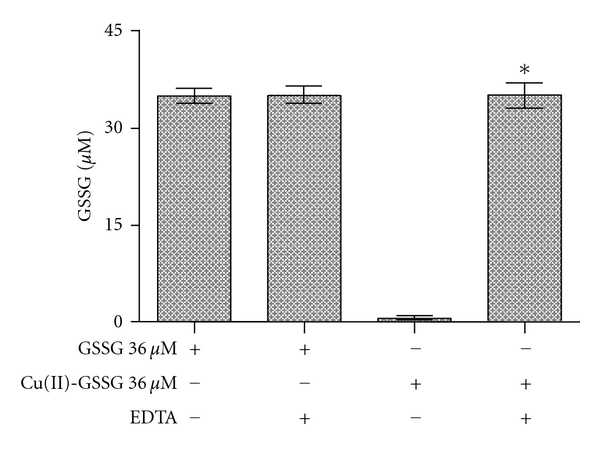
Effect of EDTA on the recovery of GSSG from the Cu(II)-GSSG complex assayed by the NADPH/glutathione reductase assay. NADPH (0.2 mM), glutathione reductase (2 U/mL), and EDTA (2 mM) were added to a solution containing GSSG (36 *μ*M) or a preformed Cu(II)-GSSG complex (36 *μ*M). The decrease in OD_340 nm_ was registered and the results expressed as *μ*M GSSG concentration. The asterisk on the bars indicate that such values are statistically different, at the level of a *P* < 0.001, from all other values.

**Figure 4:**
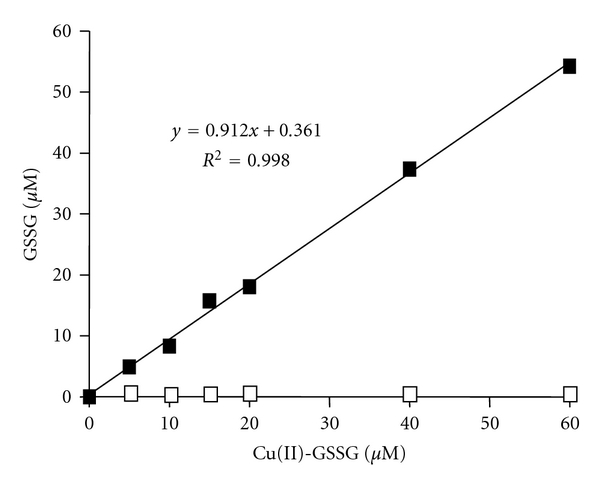
Assessment of GSSG by the EDTA-modified NADPH/glutathione reductase assay: correlation between GSSG and Cu(II)-GSSG. NADPH (0.2 mM), glutathione reductase (2 U/mL), and EDTA (2 mM) were added to solutions containing increasing concentrations of the Cu(II)-GSSG complex (5–60 *μ*M). Results are expressed as *μ*M GSSG concentration. The symbols represent: solutions containing the Cu(II)-GSSG complex (5–60 *μ*M) assayed in the presence (■) or in the absence of EDTA (□).

**Figure 5:**
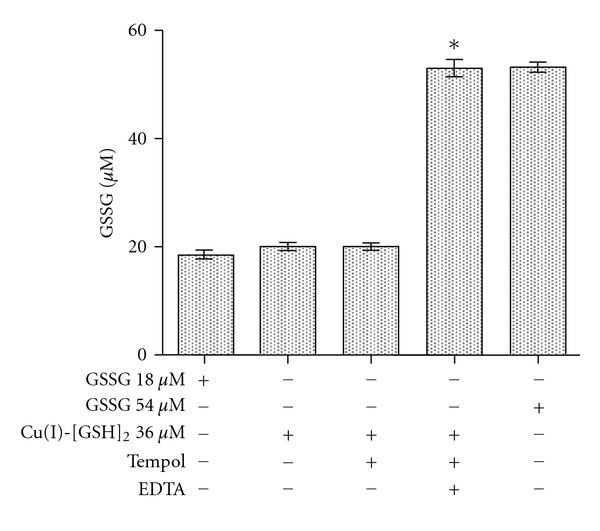
Recovery of GSSG from a Tempol-treated Cu(I)-[GSH]_2_ complex using the EDTA-modified NADPH/glutathione reductase assay. GSSG was assessed applying the NADPH/glutathione reductase assay to solutions containing pure GSSG (18 or 54 *μ*M), the Cu(I)-[GSH]_2_ complex (36 *μ*M) or a Tempol-treated Cu(I)-[GSH]_2_ complex (36 *μ*M). The assays were run both in the presence or in the absence of EDTA (2 mM). Results are expressed as *μ*M GSSG concentration. The asterisk on the bars indicates that such values are statistically different, at the level of a *P* < 0.001, from all other values.

**Figure 6:**
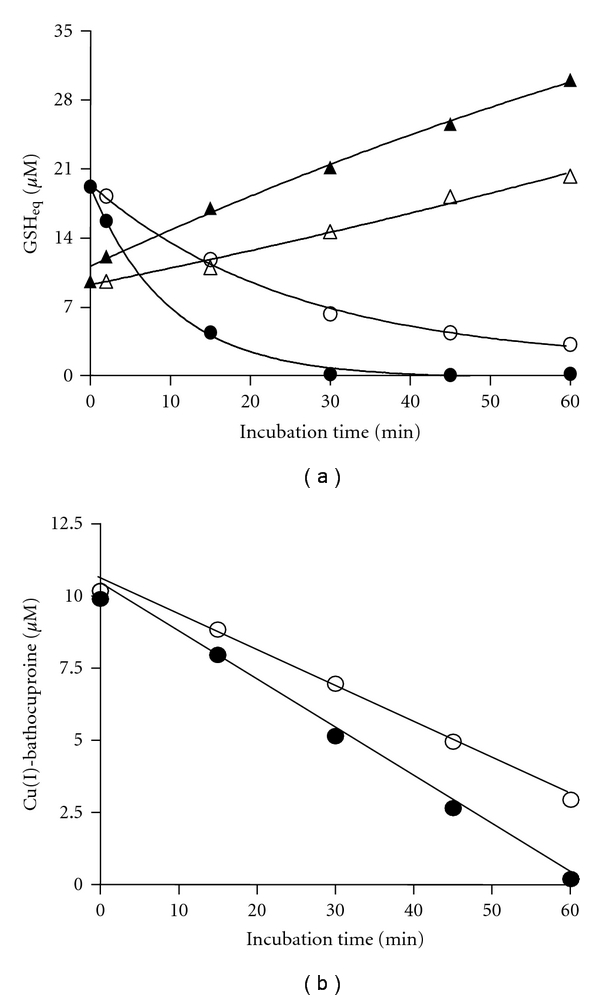
(a) Changes in the thiol-titratable groups and EDTA-releasable GSSG during the exposure of a Cu(I)-[GSH]_2_ complex to Tempol. For the determination of TTG (circles), a mixture of DTNB (24 *μ*M) plus EDTA (2 mM), and for that of GSSG (triangles), a mixture of NADPH (0.2 mM) plus glutathione reductase (2 U/mL) and EDTA (2 mM) were added to samples obtained from the incubation (0–60 min at 30°C) of the Cu(I)-[GSH]_2_ complex (10 *μ*M) both in the presence (closed symbols) or in the absence (open symbols) of Tempol (8 *μ*M). Results are expressed as GSH equivalents (*μ*M). (b) Changes in copper (I) during the exposure of a Cu(I)-[GSH]_2_ complex to Tempol. For the determination of Cu(I) (circles), bathocuproine (500 *μ*M) was added to samples obtained from the incubation (0–60 min, at 30°C) of the Cu(I)-[GSH]_2_ complex (10 *μ*M) both in the presence (closed symbols) or in the absence (open symbols) of Tempol (8 *μ*M). Cu(I) was assayed at OD_480 nm_ and the results expressed as Cu(I)-bathocuproine (*μ*M).

**Figure 7:**
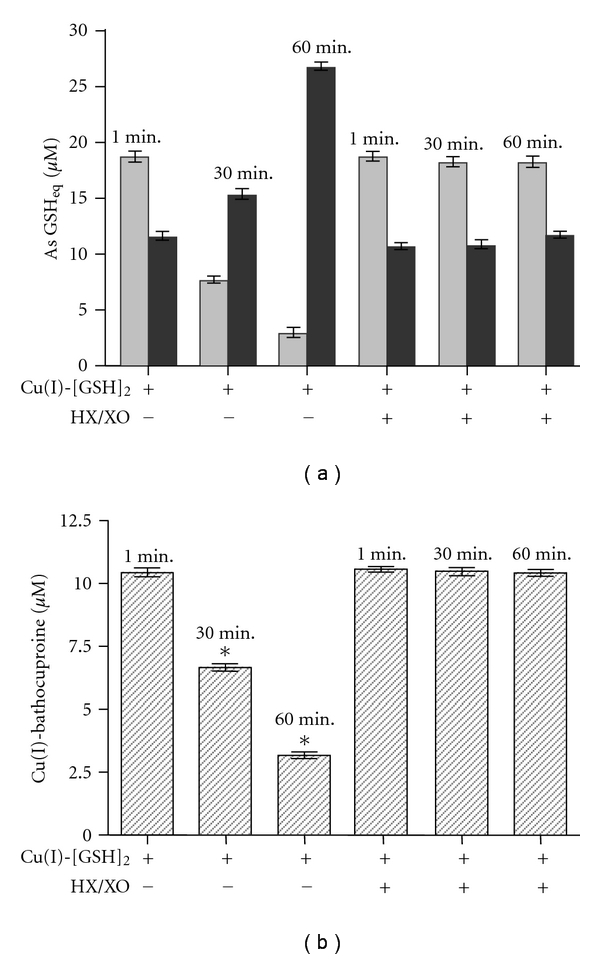
(a) Effect of the exposure of Cu(I)-[GSH]_2_ to an exogenous source of superoxide radicals on the thiol-titratable groups and EDTA-releasable GSSG. Hypoxanthine (0.2 mM) plus xanthine oxidase (XO; 2 U/mL) were added to a solution containing the Cu(I)-[GSH]_2_ complex (10 *μ*M), and the mixture was incubated for 0–60 min, at 30°C. Samples were taken from the incubation media after 1, 30, and 60 min and analyzed for their TTG (light grey bar) and EDTA-releasable GSSG (dark grey bar) levels. Results are expressed as GSH equivalents (*μ*M). (b) Effect of the exposure of Cu(I)-[GSH]_2_ to an exogenous source of superoxide radicals on the concentration of copper (I). Hypoxanthine (0.2 mM) plus xanthine oxidase (XO; 2 U/mL) were added to a solution containing the Cu(I)-[GSH]_2_ complex (10 *μ*M) and the mixture was incubated for 0–60 min, at 30°C. Samples were taken from the incubation media after 1, 30, and 60 min and analyzed for their Cu(I) concentration. Results are expressed as Cu(I)-bathocuproine (*μ*M). The asterisk on the bars indicate that such values are statistically different, at the level of a *P* < 0.001, from all other values.

Data points represent the means of at least 3 independently run experiments, each conducted in triplicate. For the sake of simplicity and since the standard deviation values represented less than 5% of the means, these were omitted from all point graphs (i.e., Figures 1, 2, 4, and 6). In the case of Figures 3, 5, and 7 (plotted as bar graphs), however, since some of the means exhibited standard deviations greater than 5%, the latter were included. When evaluated, the statistical significance of the difference between bars was assessed using the Student's *t*-test. GraphPad Prism 4 was used as statistical software.

## 3. Results and Discussion

Previous studies indicate that the interaction between GSH and Cu^2+^ ions leads to the swift formation of a 1 : 2 copper-glutathione complex, presumably Cu(I)-[GSH]_2_ (as shown in (Rx. 1)) [8, 10, 25]. In accordance with such proposed stoichiometry, a near 20 *μ*M concentration of thiol-titratable groups (TTG) can be recovered in a solution containing a freshly prepared 10 *μ*M concentration of the Cu(I)-[GSH]_2_ complex (Figure 1). However, as shown in the same figure, the stability of the thiols can be significantly affected by the sole incubation of such solution at 30°C. In fact, the level of TTG started to decline steadily along time, to reach after 60 min one third of the initial values (near 7 *μ*M). Such decline was markedly increased when the solution containing the Cu(I)-[GSH]_2_ complex was coincubated at 30°C but in the presence of Tempol (8 *μ*M). In fact, TTG levels decreased by 75% to reach a 5 *μ*M concentration after 15 min and totally disappeared after 30 min of incubation. Since Tempol has no oxidizing effect on GSH molecules (not shown), its TTG-declining effect would be attributable to its superoxide dismutase-mimetic properties [26, 27]. A similar but clearly more accelerated TTG-declining effect of Tempol was observed when the Cu(I)-[GSH]_2_ complex and Tempol were coincubated at 37°C instead of 30°C (insert to Figure 1). Under such conditions, the concentration of thiol-titratable groups was negligible already after 10 min. From the figure, it is evident, however, that the sole incubation at 37°C itself has a significant TTG-declining effect. Such “Tempol-like effect” of the temperature could be explained by the recently reported temperature-dependent increment in the rate at which the superoxide radicals generated by the complex (Rx. 2) undergo spontaneous dismutation [16, 28].

To assess whether the loss of TTG induced by Tempol arises from the oxidation of the GSH molecules bound to Cu(I) (within the complex), GSSG was quantified in samples of the above-referred Tempol-treated complex using the GSSG specific NADPH/glutathione reductase assay [22]. To imply that the 20 *μ*M loss of TTG observed in Figure 1 arises solely from the oxidation of Cu(I)-bound GSH molecules, an increment of 10 *μ*M in the concentration of GSSG would be expected. We found, however, that after incubating the complex with Tempol during 30 min (at 30°C), the concentration of GSSG assayable in the media was only 5 *μ*M (not shown). Notably, such concentration was already present at time zero, when solutions containing the freshly prepared complex were incubated in the absence or presence of Tempol. Thus, the “limited recovery” of GSSG, namely, only 5 *μ*M (instead of the sum of the initial 5 *μ*M plus the expected 10 *μ*M increment in GSSG) suggests that the actual loss of TTG involves the generation of GSSG molecules that cannot be directly quantified by the NADPH/Glutathione reductase assay. 

Recently, using EPR and NMR techniques, we observed that incubation of Cu(I)-[GSH]_2_ with SOD leads to the total disappearance of such complex and to a concomitant accumulation of a new complex which was identified as Cu(II)-GSSG [16]. Thus the above-referred only partial recovery of TTG in the form of GSSG from the Tempol-treated Cu(I)-[GSH]_2_ complex could be explained by the assumption that all the newly formed GSSG molecules end up bound to Cu(II) ions. In such form, GSSG molecules would be precluded from being directly quantified by the NADPH/Glutathione reductase assay. In view of the latter consideration, we took advantage of the early reported ability of the Cu(II)-GSSG complex to absorb at 625 nm [17]. As shown in Figure 2, the incubation of Cu(I)-[GSH]_2_ (5 mM) with Tempol (at 37°C) led to a time-dependent, sustained, and linear formation of Cu(II)-GSSG. After 90 min, near 80% of the initial concentration of Cu(I)-[GSH]_2_ was converted into Cu(II)-GSSG. Likewise seen in the experiments depicted in the insert to Figure 1, in which TTG were assessed, we observed that the sole incubation of Cu(I)-[GSH]_2_ at 37°C led, after 90 min, to its oxidation by near 30% (Figure 2). Thus, conditions which accelerate the dismutation of the superoxide anions generated in (Rx. 2) (whether Tempol-mediated or temperature-induced) appear to favor the irreversible conversion of Cu(I)-[GSH]_2_ into Cu(II)-GSSG ((Rx. 2) and (Rx. 3))

(Rx. 3) $$\text{IOC}+2{{\text{O}}_{2}}^{∙-}\underset{T^{{}^{\circ}}}{\overset{\text{Tempol}}{\to }}\text{Cu}\left(\text{II}\right)\text{-GSSG}+{\text{H}}_{2}{\text{O}}_{2}+{\text{O}}_{2}$$

Although measuring the OD at 625 nm seems to be useful to assess the occurrence and formation of Cu(II)-GSSG, its application is limited to the estimation of only millimolar concentrations of such complex (*ε* = 60 M^−1^ cm^−1^) [17]. Thus, in order to assess the oxidative (Tempol-mediated) conversion of micromolar concentrations (expected to occur in biological systems) of Cu(I)-[GSH]_2_ into Cu(II)-GSSG, we conducted experiments aimed to quantify those GSSG molecules that are bound to Cu(II). As already suggested, such molecules do not appear to be quantifiable by the NADPH/glutathione reductase assay.  Figure 3 shows the levels of GSSG obtained upon applying such assay to samples of both free GSSG molecules and a preformed Cu(II)-GSSG complex. As shown, virtually no GSSG molecules (less than 1%) were recovered in the samples containing Cu(II)-GSSG (36 *μ*M). The latter was, however, totally reversed when the NADPH/glutathione reductase assay was performed in the presence of EDTA. In fact, under such assay conditions, a 100% of the GSSG contained in the Cu(II)-GSSG complex was recovered (Figure 3). As such, EDTA did not affect GSSG recovery from a solution containing solely the free disulfide. Thus, the full recovery of GSSG from a solution containing Cu(II)-GSSG suggests that EDTA makes GSSG molecules freely available to react with the reductase. Both EDTA [29] and GSSG [30] are able to chelate Cu(II). The stability constant of Cu(II)-EDTA (*K* = 6.3 × 10^18^) [29] is, however, five orders of magnitude higher than that of Cu(II)-GSSG (*K* = 5.6 × 10^13^) [30]. Thus, in the presence of EDTA, Cu(II) ions are most likely to be efficiently removed from Cu(II)-GSSG. As shown in Figure 4, applying the NADPH/glutathione reductase plus EDTA assay to various concentrations of a preformed Cu(II)-GSSG complex allowed the indirect and total quantification of the complex. Linearity for such assay conditions was established within the 5–60 *μ*M range of concentrations (Figure 4). 

Taking advantage of the ability of EDTA to release the GSSG bound to Cu(II), the formation of Cu(II)-GSSG in a Tempol-treated Cu(I)-[GSH]_2_ solution was quantitatively re-assessed. To distinguish between free and Cu(II)-bound GSSG molecules, the levels of the disulfide were assessed both in the absence and in the presence of EDTA in the assay media, respectively (Figure 5). When a 36 *μ*M Cu(I)-[GSH]_2_ solution (prepared by mixing 36 *μ*M of Cu^2+^ plus 108 *μ*M of GSH) was assayed in the absence of EDTA, an 18 *μ*M concentration of GSSG was recovered as free disulfide. Such concentration fully corresponds to that expected to occur if, as in (Rx. 1), GSH oxidation had arisen exclusively from the initial reduction of the Cu^2+^ ions needed to form the Cu(I)-[GSH]_2_ complex. An identical result was found when GSSG was assayed, also in the absence of EDTA, in a Tempol-treated Cu(I)-[GSH]_2_ solution (Figure 5). In turn, when the latter solution was assayed in the presence of EDTA, the total concentration of GSSG recovered raised from 18 *μ*M to 54 *μ*M. The latter value corresponds exactly to the sum of 18 *μ*M (occurring as free GSSG) plus 36 *μ*M (of GSSG released by EDTA from the Cu(II)-GSSG complex). According to these results, it would be possible to estimate the concentration of GSSG bound to Cu(II), namely, that of the Cu(II)-GSSG complex, by subtracting the concentration of GSSG assayed in the absence from that estimated in the presence of EDTA.  Figure 6(a) shows the results from applying this EDTA-dependent GSSG recovery assay to Cu(I)-[GSH]_2_-containing solution incubated in the absence or in the presence of Tempol. While the descending curves of Figure 6(a) depict the loss of TTG that affect the complex along its 60 min of incubation (data from Figure 1), the ascending ones show the corresponding increments in the EDTA-releasable GSSG. In both cases, results are expressed as GSH equivalents for comparative purposes. Interestingly, regardless of whether Tempol was added to the complex, the loss of TTG was along time accompanied by a concomitant but only quantitatively-partial gain of EDTA-releasable GSSG (Figure 6(a)). For instance, in the case of the Tempol-treated solution, the complex underwent after 30 min a total loss of its TTG, decreasing from 20 *μ*M to 0 *μ*M GSH eq. In turn, the gain in GSSG was only half of such loss, as manifested by an increase in GSH eq. from 10 *μ*M to 20 *μ*M. Thus, after 30 min, only 50% of the TTG that had disappeared could be accounted for by the formation of EDTA-releasable GSSG. Comparatively, after 60 min of incubation, the loss of GSH was totally accounted for by the estimated increment in GSSG. In fact, the concentration of GSSG raised from 10 *μ*M to 30 *μ*M GSH eq., leveling the 20 *μ*M of GSH eq. that had been lost earlier as TTG. The lack of correspondence between the GSH eq. lost as TTG and those gained as EDTA-releasable GSSG seen after 30 min could be explained assuming that Tempol accelerated oxidative changes in the Cu(I)-[GSH]_2_ molecule which lead to the formation of the an “intermediate oxidized complex”. Presumably, the structure of such intermediate would feature some form of thiols that are not reactive to DTNB (i.e., not titratable as TTG) or disulfides that are not susceptible to be quantified by the EDTA/NADPH/glutathione reductase assay (different from Cu(II)-bound GSSG molecules). The formation of such intermediate (referred to as IOC in (Rx. 2)) would precede and potentially lead to the formation of the Cu(II)-GSSG complex. 

To further our understanding on the changes involved in the oxidative conversion of Cu(I)-[GSH]_2_ into Cu(II)-GSSG, we investigated whether exposure of the former complex to Tempol involves also time-dependent changes on the copper bound to GSH. As shown in Figure 6(b), Tempol led to a time-dependent decrease in the concentration of Cu(I), as assessed by the Cu(I)-bathocuproine assay [24]. Interestingly, after 30 min of incubation, almost 50% of the Cu(I) still remained as such. The latter is in sharp contrast with the fact that at the indicated time, 100% of the TTG had already disappeared (data in Figure 6(a)). The lack of correspondence between the magnitude of the oxidative changes that affect Cu(I) and GSH within the Cu(I)-[GSH]_2_ complex suggests that after 30 min, the Cu(I) that remains as such would no longer form part of such complex. As for the TTG, the extent of the disappearance of Cu(I) shown in Figure 6(b) was also accelerated by Tempol. Both changes support the concept that removal of superoxide from (Rx. 2) favors the conversion of the putative intermediate into Cu(II)-GSSG (Rx. 3) and are in line with data from Figure 2, where Tempol was shown to accelerate the formation of such complex. 

Furthermore, when the incubation of Cu(I)-[GSH]_2_ plus Tempol was extended to 60 min, time at which 100% of the Cu(I) had disappeared (Figure 6(b)), a 100% of the TTG that was lost at such time could be recovered as EDTA-releasable GSSG (Figure 6(a)). The latter changes would indicate that after 60 min, removal of superoxide by Tempol secures the total conversion of Cu(I)-[GSH]_2_ complex (and its oxidized intermediate) into Cu(II)-GSSG. Interestingly, such conversion also takes place even in the absence of Tempol, but at a much slower rate, suggesting that superoxide radicals generated in (Rx. 2) also undergo spontaneous dismutation under such incubation condition (30°C). 

Based on all the above presented results, we propose that removal of superoxide anions from (Rx. 2) (whether spontaneous or Tempol-induced) leads time dependently to the oxidative disappearance of GSH (evidenced through a loss of TTG and formation of EDTA-releasable GSSG) and Cu(I) from Cu(I)-[GSH]_2_. Based on such contention, we pursued additional experiments to investigate the superoxide-dependent reversibility of (Rx. 2). Specifically, we evaluated whether increasing the flow of superoxide in such reaction, through an exogenous source of such radicals, could slow the rate at which the Cu(I)-[GSH]_2_ complex undergoes the oxidation of GSH and Cu(I). Thus, a solution containing Cu(I)-[GSH]_2_ was incubated (during 1, 30 or 60 min) in the absence or in the presence of hypoxanthine plus xanthine oxidase (HX/XO).  Figure 7(a) depicts the changes in the TTG and the EDTA-releasable GSSG levels, expressing both changes in terms of GSH equivalents. As seen, the incubation of Cu(I)-[GSH]_2_ plus HX/XO prevented by near 70% and by 72% the loss of TTG observed otherwise at 30 and 60 min, respectively. Regarding the formation of EDTA-releasable GSSG, the presence of HX/XO totally prevented the time-dependent increment that the complex underwent in the absence of the additional source of superoxide. Consistent with these results, the HX/XO mixture also totally prevented the time-dependent loss of bathocuproine assayable Cu(I) ions (Figure 7(b)). Therefore, data from Figures 7(a) and 7(b) support the concept that (Rx. 2) is indeed reversible and suggest that preventing the removal of the superoxide anions generated in such reaction is key to maintain its equilibrium and to secure that in the same reaction, the postulated IOC, instead of shifting towards the formation of the Cu(II)-GSSG complex (Rx. 3), would serve primarily to regenerate the Cu(I)-[GSH]_2_ complex (Rx. 2). Most cells contain significant amounts of SOD and a number of other molecules susceptible to react with superoxide, like GSH and ascorbate which could reach millimolar concentrations within the cells. In addition to the latter superoxide-reacting molecules, ferritin, the major intracellularly occurring iron-storing protein was recently reported to react with the superoxide anions generated by the Cu(I)-[GSH]_2_ complex [31]. Thus, it could be speculated that due to the presence of various superoxide removing or reacting molecules, the Cu(I)-[GSH]_2_ complex might occur within cells, not only as such but also as Cu(II)-GSSG, its end-oxidation product.

## 4. Conclusions

Removal of superoxide anions from a solution containing the Cu(I)-[GSH]_2_ complex led to losses in GSH molecules that were of greater magnitude than those affecting the Cu(I) metal. Since the loss of TTG was only partially accounted for by the amount of Cu(II)-GSSG molecules accumulated, the formation of an “intermediate oxidized complex”, whose thiols are neither reactive towards DTNB nor susceptible to be quantified by the EDTA/NADPH/glutathione reductase assay, can be postulated. Since the oxidative drops in GSH and Cu(I) were totally prevented by the exposure of the Cu(I)-[GSH]_2_ complex to an external source of superoxide, it is suggested that the generation of superoxide in (Rx. 2) would imply an obliged formation of the above-referred intermediate. Results support the contention that the subsequent oxidation of such intermediate into Cu(II)-GSSG or its reduction back into Cu(I)-[GSH]_2_ can be favoured by either the removal or the addition of superoxide anions to the media, respectively.
